# Complete Genomic Sequence of a Novel Reassortant H4N2 Avian Influenza Virus Isolated from Domestic Ducks in Jiangsu, China

**DOI:** 10.1128/genomeA.00091-13

**Published:** 2013-03-14

**Authors:** Qingqing Zhao, Qunhui Li, Lei Zhong, Min Gu, Jie Zhu, Guo Zhao, Chaoyang Chen, Xiaoquan Wang, Xiaowen Liu, Xiufan Liu

**Affiliations:** a Animal Infectious Disease Laboratory, College of Veterinary Medicine, Yangzhou University, Yangzhou, Jiangsu, China; b Ministry of Education, Key Lab for Avian Preventive Medicine, Yangzhou University, Yangzhou, Jiangsu, China

## Abstract

Here, we report the complete genomic sequence of a novel reassortant H4N2 influenza virus isolated from domestic ducks in the Jiangsu province of China in 2011. Phylogenetic analysis showed that all the viral genes except for hemagglutinin (HA) were highly homologous to the clade 2.3.4 H5N2 viruses. The data suggest that genetic reassortment occurred between H4 and H5N2 avian influenza viruses, which highlights the role of domestic poultry as a reassortment vessel in China.

## GENOME ANNOUNCEMENT

Avian influenza viruses (AIVs) are negative-sense RNA viruses that belong to the *Orthomyxoviridae* family (1, 2). Based on serologic and genetic analyses, AIVs are classified into 16 hemagglutinin (HA) and 9 neuraminidase (NA) subtypes (3). In the past few decades, H4 AIVs, which generally produce asymptomatic infections, have been isolated from domestic ducks (4) and pigs (5). In addition, H4 viruses may have the ability to cross the species barrier to infect humans (6). Therefore, the H4 subtype of AIVs that circulates in poultry should not be ignored. Strengthening the investigation of H4 viruses is important for the study of the evolution and variation of AIVs.

In January 2011, a novel recombinant H4N2 influenza virus, designated strain A/duck/Jiangsu/1-15/2011 (H4N2), was isolated from a domestic duck in a live poultry market in the Jiangsu province of eastern China. Here, we analyzed the complete genome sequence of the strain to investigate its molecular evolution and reassortment characteristics. The full genes of the strain were sequenced with an ABI 3730 genetic analyzer (Applied Biosystems).

The complete genome of the strain consists of eight segments, including PB2, PB1, polymerase acidic (PA), HA, nucleoprotein (NP), NA, matrix (M), and nonstructural (NS) proteins. The full lengths of the segments are 2,341, 2,341, 2,233, 1,738, 1,565, 1,467, 1,027, and 890 nucleotides, respectively. The amino acid sequence at the cleavage region of the HA gene is PEKASR/GLF, which is typical for low-pathogenicity AIVs. The amino acid residues at the receptor binding site in the HA protein are Q226 and G228 (H3 numbering), which indicates its avian-like receptor binding preference. The strain has five potential *N*-glycosylation sites at positions 18, 34, 178, 310, and 497 in the HA protein, and seven sites at positions 61, 69, 70, 146, 200, 234, and 402 in the NA protein. The PB2 protein possessed E627 and D701, which is characteristic of avian influenza virus. Furthermore, no deletions were found in the NA and NS genes.

Phylogenetic analysis revealed that all eight genes belong to the Eurasian lineage. Complete sequence analysis showed that with the exception of the HA gene, which had the highest nucleotide sequence identity with that of strain A/wild duck/Korea/CSM4-28/2010 (H4N6) (98.8%), the other seven genes (PB2, PB1, PA, NP, NA, M, and NS) were all mostly related to the previously reported novel reassortant highly pathogenic avian influenza (HPAI) H5N2 viruses A/duck/Eastern China/1111/2011 (>99.0%) and A/goose/Eastern China/1112/2011 (98% to 99%). It is noteworthy that those two H5N2 viruses were derived from the clade 2.3.4 HPAI H5N1 viruses by incorporating another N2 gene (7). Therefore, the virus A/duck/Jiangsu/1-15/2011 (H4N2) might be a further reassortant in which the HA gene originated from H4 subtype AIV and the remaining seven genes came from the HPAI H5N2 AIV. The detection of this novel reassortant H4N2 virus with its potential threats to both animal and public health highlights the genetic reassortment between viruses of different subtypes and the evolution of AIV in China.

In conclusion, these data emphasize the necessity to strengthen the surveillance and epidemiological investigation of AIVs in China.

### Nucleotide sequence accession numbers.

The complete genomic sequence of A/duck/Jiangsu/1-15/2011 (H4N2) was deposited in GenBank under the accession no. KC282876 to KC282883.
